# Molecule Formats of B-Cell Targeting Biologics: Applications in Autoimmune Disease Treatment and Impacts on Manufacturability

**DOI:** 10.3390/pharmaceutics17040495

**Published:** 2025-04-08

**Authors:** Yueming Qian, Tamer I. Mahmoud

**Affiliations:** 1 Pre-Pivotal Drug Substance Technologies, Amgen Inc., Rockville, MD 20850, USA; 2 Discovery Research, Amgen Inc., Rockville, MD 20850, USA; tmahmoud@amgen.com

**Keywords:** autoimmune disease, biologics manufacturability, molecule format, therapeutic target

## Abstract

The targeting of B-lymphocyte cells has emerged as one of the most pivotal strategies in the management of autoimmune diseases. This review provides an overview of protein therapeutics illustrating their direct and indirect effects on B-cells using different molecule formats. The design and format of these molecules influence their mode of action and affect their manufacturing strategies. Manufacturability should be assessed at an early stage and continuously through collaboration between discovery and development teams. Scalability evaluations should encompass not only process development and facility compatibility but also cell line development. Examples of format-specific manufacturability of biologics are reviewed to offer general insights into enhancing productivity and quality in a cost-effective manner.

## 1. Introduction

The immune system includes the innate and adaptive subsystems, which work together to defend against infection and disease. In autoimmune diseases, the immune system may malfunction, erroneously targeting its own cells, tissues, and organs. The NIH Autoimmune Disease Coordinating Committee reports that there are at least 80 types of chronic and often disabling autoimmune disorders, affecting about 24 million people in the United States (https://dpcpsi.nih.gov/sites/default/files/NIH-Triennial-Report-FY-2016-2018_Final508.pdf, accessed on 3 April 2025). The root cause remains poorly understood, but an overactive response is frequently observed in the immune network, involving humoral, cellular components, or both.

B-cells are vital for humoral immunity, but they can become pathogenic in autoimmunity [1]. Autoreactive B-cells may produce autoantibodies against the body’s tissues, present self-antigens to T-cells, or generate inflammatory cytokines. Due to their role in autoimmune disease initiation and progression [2], B-cells have become key therapeutic targets. Strategies to target B-cells vary based on the disease mechanism and B-cell functions [3]. B-cell depletion effectively treats pemphigus and neuromyelitis optica spectrum disorder (NMOSD) [4] but not for other autoimmune diseases, such as systemic lupus erythematosus (SLE) [3]. The variability in efficacy may be associated with the breadth and depth of pathogenic B-cell depletion and the diversity of B-cell functions being a driver of pathophysiology in these heterogeneous diseases.

There are different methods to target B-cells [5], such as cell-based and biologics-based therapeutics. Targeting B-cells by biologics can be direct or indirect. Direct depletion is achieved by targeting surface molecules like CD20, CD19, and B-cell-activating factor receptors (BAFF-R) using monoclonal antibodies. Indirect depletion involves blocking survival cytokines or regulators of activation and differentiation. BAFF/BAFF-R interaction promotes survival gene expressions in B-cells and plasma cells where BAFF-R is a direct target and BAFF is an indirect target. CD40 and CD40L are another pair of direct and indirect B-cell targets; blocking their interaction inhibits B-cell gene transcription, cell growth, and differentiation into memory B cells and plasma cells. Other modalities, such as Chimeric Antigen Receptor (CAR) T-cell therapy, have also been explored to target B-cells; however, the following sections will only discuss protein therapeutics that target/interact with B-cells directly or indirectly. The focus will be on the impacts of biologics formats on their mode of action and manufacturability. This topic, yet to be reviewed, is crucial for advancing biologics development from discovery through to manufacturing.

## 2. Different Molecule Formats and Features Applied in Biologics Development for Autoimmune Disease Treatments

Previous review articles have collectively provided a good reference for B-cell targeting biologics from different perspectives [6,7,8,9]. Table 1 summarizes the related contents with additional molecules, new updates, and the highlights of molecule formats and features. Six representative products are discussed relating to different molecule formats and features applied in biologics development and their mode of action in the associated diseases.

### 2.1. Ocrelizumab and Ofatumumab—Next Generation Anti-CD20 Monoclonal Antibodies (mAbs)

Both ocrelizumab and ofatumumab, as examples of next-generation B-cell targeting mAbs, were designed to reduce immunogenicity with the former humanized and the latter fully human. Ocrelizumab binds an epitope that overlaps with rituximab’s binding site and offers enhanced antibody-dependent cell-mediated cytotoxicity (ADCC) [7] whereas ofatumumab specifically recognizes an epitope that encompasses both the small and large extracellular loops of CD20 and has a more effective complement-dependent cytotoxicity (CDC) induction and killing target cell capacity [32]. Ocrelizumab was first approved in the US for the treatment of patients with relapsing–remitting multiple sclerosis (RRMS) and primary progressive multiple sclerosis (PPMS) in 2017 [33], which led to B cell depletion being a mainstay of treatment for MS, and the first therapy specifically for PPMS [34]. In addition to the approvals for RRMS, PPMS, and secondary progressive MS (SPMS) [35], Ofatumumab is also under clinical investigation for RA.

### 2.2. Inebilizumab—Afucosylated Anti-CD19 mAb

Developed by Viela Bio and acquired by Horizon Therapeutics and more recently by Amgen, inebilizumab is a humanized IgG1 mAb targeting the extracellular loop of CD19 [36,37], It initially received FDA approval in 2020 for the treatment of NMOSD in adult patients who are seropositive for immunoglobulin G (IgG) autoantibodies against aquaporin-4 (AQP4) [4]. Since then, the authorization for access to this biologic drug has been quickly expanded to include NMOSD patients in Japan in 2021, in Germany and France in August 2022, and in Brazil in December 2022 [38]. As an afucosylated IgG1, inebilizumab has a 10-fold higher binding affinity to human Fcγ receptor (FcγR) IIIA and achieves quick and potent B-cell depletion through ADCC [36]. Further clinical data showed that the beneficial outcomes could be maintained for more than 4 years in terms of the reductions in NMOSD attacks [39] and in AQP4-IgG titers. Other indications such as kidney transplant desensitization, myasthenia gravis, and IgG4-RD are also under clinical evaluation [4]. Phase 3 clinical trial for the treatment of IgG4-RD was a randomized, double-blind, and placebo-controlled study at 80 sites in 22 countries. Compared to the placebo for its primary endpoint, this novel and the steroid-sparing trial showed a statistically significant 87% reduction in the risk of IgG4-RD flare for 52 weeks; all key secondary endpoints including annualized flare rate and treatment-free and corticosteroid-free complete remission were all met [14]. In a Phase 3 trial in myasthenia gravis (NCT04524273), inebilizumab met its primary endpoint, with a statistically significant change from baseline in Myasthenia Gravis Activities of Daily Living (MG-ADL) score (−4.2) compared with placebo (−2.2) (difference: −1.9, *p* < 0.0001) at Week 26 for the combined study population, and demonstrated continued improvement through this period of time (https://wwwext.amgen.com/newsroom/press-releases/2024/10/amgen-presents-positive-phase-3-data-for-uplizna-inebilizumabcdon-in-generalized-myasthenia-gravis-gmg-at-aanem-2024, accessed on 15 March 2025).

### 2.3. Belimumab—mAb Indirectly Targeting B Cells

Belimumab is a fully human mAb that does not directly target B-cells but neutralizes the biologically active soluble form of BAFF and in turn, blocks the binding of this cytokine stimulator to its receptor on the involved B-cells [20]. It has been found that BAFF overexpression induces autoreactive B cells to increase autoantibody levels under autoimmune conditions [40]. Inhibition of B-cell proliferation and differentiation by belimumab represents a treatment option for adult patients with seropositive active SLE (especially musculoskeletal and cutaneous disease). Following the FDA approval of belimumab in 2011, which was the first biologic approved for SLE and the first new lupus drug in more than 50 years [20], BAFF was established as a molecular target for further therapeutic developments. Telitacicept is an Fc fusion protein that contains TACI amino acids 13-118 of the extracellular domain, which binds to and neutralizes BAFF and APRIL. It received approval for SLE in China in 2021 [29]. Conversely, tabalumab is a humanized monoclonal antibody designed to have a broader blocking effect by neutralizing both soluble and membrane-bound BAFF. However, it did not achieve the expected clinical results in relapsing multiple sclerosis [41,42]. Furthermore, atacicept is another TACI-Ig fusion protein that binds to and blocks BAFF and APRIL [41]. Blisibimod, an Fc-conjugated peptibody, was developed as a specific inhibitor with high avidity antagonizing both soluble and membrane-bound BAFF [43]. Nonetheless, neither atacicept nor blisibimod met clinical expectations in SLE treatment, highlighting the challenges within this field [20].

### 2.4. Ianalumab (VAY736)—mAb Directly Targeting B Cells

Ianalumab is a fully human mAb that specifically targets BAFF-R to lyse B-cells through ADCC and interrupt B-cell maturation, proliferation, and survival via blocking BAFF-mediated signaling [44]. It has been explored for potential therapeutic effects in various autoimmune conditions including MS, SLE, LN, and Sjögren’s disease, and more recently in patients with diffuse cutaneous system sclerosis. Subcutaneous administration of ianalumab in a randomized, double-blind, placebo-controlled study was well-tolerated and resulted in clinical improvements in Sjögren’s patients with statistically significant dose–response for overall disease activity [21].

### 2.5. Dazodalibep (HZN4920/AMG611)—HSA-Fusion Protein Antagonizing CD40L

Dazodalibep is a novel CD40 ligand (CD40L) antagonist. It is human serum albumin (HSA)-fusion protein possessing two Tn3 scaffolds [27], derived from the third fibronectin type III domain of human tenascin-C. Tn3 contains Ig-like folds, structurally analogous to antibody complementarity-determining regions (CDR). The Tn3 scaffolds of dazodalibep have been engineered to confer binding specificity to CD40L [28]. Lacking an Fc domain, dazodalibep does not lead to platelet aggregation or activation [28]. As an indirect and non-depleting B-cell modulator, dazodalibep blocks CD40L on T cells interacting with CD40-expressing B cells and disrupts the overactivation of the CD40 co-stimulatory pathway. Its clinical trials for several autoimmune diseases, such as Sjögren’s disease, kidney transplant rejection, and rheumatoid arthritis, are ongoing or have been completed. In addition to the positive data from the Phase 2 trial in patients with RA [26], the results from the Phase 2 trial for patients with Sjögren’s syndrome met the primary endpoint and led to the design of a Phase 3 program [45].

### 2.6. PRV-3279—Bispecific Antibody

PRV-3279 (formerly MGD010) is a humanized dual-affinity-retargeting (DART) bispecific molecule. This antibody targets B-cell surface proteins CD32B and CD79B simultaneously, developed by MacroGenics and licensed by Provention Bio. CD32B (FcγRIIB) is a low-affinity inhibitory receptor for IgG [46], while CD79B is part of the B cell receptor complex. Targeting CD79B alone has shown efficacy in treating autoimmune diseases in animal models [47]. Crosslinking CD32B and CD79B enhances downregulation of B-cell receptor signaling [48]. This non-depleting B-cell modulating bispecific drug aims to treat lupus and other autoimmune diseases and is currently in Phase 2a clinical trials for moderate-to-severe SLE patients (NCT05087628) [31].

## 3. Impact of Molecule Format on Manufacturability

**Figure 1:**
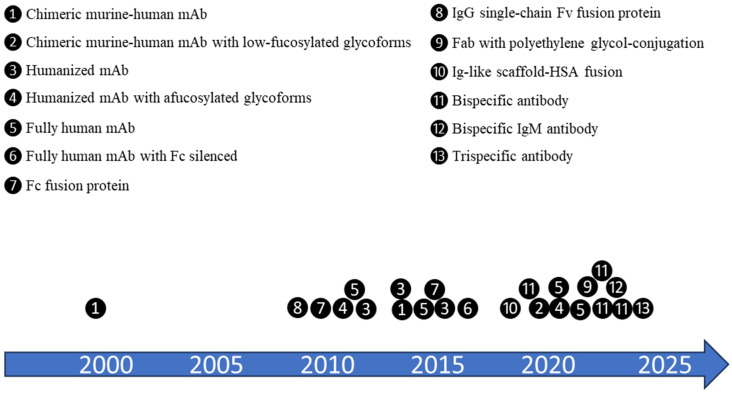
Trend in molecule formats of B-cell-targeting biologics launched or clinically trialed for autoimmune diseases. With a better understanding of the field and technological advancement, more diversified formats and enhanced features have been developed with time. Many molecules have been proven successful in oncology and are being introduced for autoimmune disease treatment [10,17]. Approximate timeline in the diagram is for the time when those biologics entered the clinical trials for autoimmune indications. ❶ = rituximab, SM03; ❷ = ublituximab; ❸ = veltuzumab, epratuzumab; BI 655064; ❹ = ocrelizumab, inebilizumab; ❺ = ofatumumab, daratumumab, belimumab, ianalumab; ❻ = iscalimab; ❼ = telitacicept, atacicept; ❽ = TRU-015; ❾ = dapirolizumab pegol; ❿ = dazodalibep; ⓫ = PRV-3279, mosunetuzumab, obexelimab, blinatumomab; ⓬ = imvotamab; ⓭ = PIT565.

While the most common type of molecule format used in B-cell targeting biologics is monoclonal antibody (mAb), other molecules with more diversified formats and enhanced features have been developed over the years (Figure 1). Many biologics were originally developed for cancer treatments and have since been repurposed for autoimmune diseases [10,17]. However, each new format presents its own set of challenges during the development and scale-up of the manufacturing process and some of them may need special considerations for manufacturability assessment and process development.

### 3.1. Molecule Format Selection and Process Development

**Figure 2:**
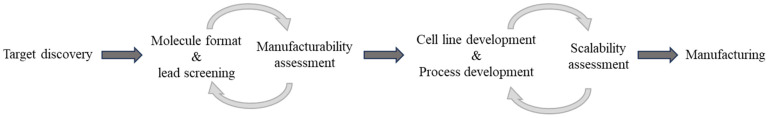
General workflow for advancing biologics development from discovery through to manufacturing. Different molecule formats may have different impacts on process development and manufacturing. Continued assessment and optimization may iteratively be needed for the development of biologics with a complex format such as bispecifics.

Manufacturability is a critical consideration to ensure the ability to manufacture a product with the desired quality at an optimized cost. In the context of biologics discovery and development (Figure 2), manufacturability assessment should be performed at an early stage through collaboration between discovery and development teams; continued evaluation and optimization are iteratively needed to improve molecule format selection, molecule design and may extend through to the entire product lifecycle management. Such reiteration could lead to “next generation” molecule design [49]. The development and commercialization of next-generation anti-CD20 antibody therapies are typical examples of these efforts [7,33]. Platform approach using standard mAb processes can be a good starting point, but off-platform methods and further optimization are often needed to ensure high-quality and cost-effective manufacturing. For example, the production of afucosylated monoclonal antibodies may require the use of specialized host cells to achieve the desired product quality attributes.

#### 3.1.1. Afucosylated mAb

Antibody glycosylation changes can significantly modulate its effector function [50]. When B-cell depletion is an intended effect, afucosylated glycoform is desirable for a therapeutic mAb, where afucosylation strongly increases IgG affinity to FcγRIIIa and thus ADCC activity [37,51,52]. Controlling glycoforms in manufacturing is challenging. Optimizing cell culture conditions can involve using small molecules to inhibit recombinant protein fucosylation [53]. However, a more efficient approach is using host cell lines with altered fucosylation processing (Table 2). These hosts may lack the ability to add fucose during glycosylation or have a shunted GDP-fucose pathway. As an example of FDA-approved afucosylated mAb drugs, UPLIZNA is produced by a proprietary FUT8^−/−^ CHO cell line [36,37]. In addition to these CHO cell lines, other host cells for afucosylated mAb productions are also reported; rat hybridoma YB2/0 cells (ATCC^®^ CRL1662™) intrinsically have lower levels of the FUT8 mRNA [54,55] and EB66 (derived from duck embryonic stem cells) have naturally reduced fucose content in the cells [52].

#### 3.1.2. Fusion Protein

Since the approval of the etanercept for RA in 1998 [63], fusion proteins have emerged as one of the most used molecule formats in autoimmune disease treatments. However, their unique structures that are often derived from different cellular locations or cell types may lead to expression issues. Fusion protein heterogeneity in isoelectric point, charge densities, and hydrophobicity could further pose challenges in manufacturing. The linkers between individual modules and their orientation are typically designed based on functional requirements. However, these elements must undergo rigorous evaluation for structural stability and quality attributes to ensure manufacturability. This initial assessment is crucial for effectively minimizing challenges related to structural integrity. For example, O-glycosylation site elimination in linker regions was found to confer Fc fusion proteins with more homogenous glycans and less aggregative propensity [64]. Rearrangement of the disulfide bonding pattern was demonstrated to not only reduce aggregate formation but also improve pharmacokinetic properties [65].

Fusion protein glycoforms can be more complex than standard mAbs, necessitating optimization of medium compositions. Bioreactor conditions such as temperature shifts and harvest timing must also be evaluated for their impact on glycan attributes [66,67]. Medium optimization for glycosylation and sialylation can be achieved by adjusting key component concentrations [68,69] or adding specific additives [70,71]. Hydrocortisone and dexamethasone enhance Fc-fusion protein sialylation [70], while LongR3, an insulin-like growth factor-I analog, increases sialic acid content and reduces asialylated N-glycan species [71].

Fusion proteins may form aggregates during upstream cultivation and downstream purification. Disulfide bond-containing fusion protein aggregation can be minimized by balancing redox equivalents in media and culture conditions [72] or regulating glutathione reductase activity by glucocorticoid receptor agonists [73]. The pH dependency of fusion domain interactions and conformational stabilities, where a low pH may induce aggregate formation, would require downstream processing to avoid acidic elution from a capture column [74] and the use of low pH for virus inactivation.

#### 3.1.3. Bispecific Antibody

Bispecifics can generally be manufactured using the same process as those for mAbs due to their structural similarities. However, variations in module formats can lead to differences in bispecifics. The manufacturing challenges include mispairing, high aggregation, and fragment formation, which necessitate early identification of sequence liabilities and the associated expression systems to ensure the correct assembly of different polypeptide chains [75]. Domain crossover, Fc heterodimerization, and common light chain strategies in protein engineering have been developed to design bispecifics with a lower tendency for mispairing and enhanced recombinant product stability.

A high-producer cell line that pairs the desired heterodimer is probably the key to allowing standard upstream and downstream processes to be utilized for bispecific production. For cell line development, titrations of separate gene constructs for transfection can increase the likelihood of correct protein assembly. Alternatively, different strengths of promoters should be used if all genes of interest are built into a single expression vector. Titer assay that measures the desired heterodimer form should be applied for rigorous clone screening.

As with standard mAb production, optimization of medium composition and culture conditions can affect product quality. To minimize aggregate formation and maximize the yield of a desired heterodimer, attention would particularly be paid to balancing redox reactions in media such as fine-tuning the concentrations of cysteine and metal ions [76]. In combination with closely monitoring overall quality attributes, temperature shifting is one of the most used strategies to improve process robustness and reduce product structural-related impurity formation [77]. To facilitate the removal of unwanted homodimers, the resin MabSelect SuRe™ pcc that has a small bead size and decreased binding avidity can be applied to the protein A matrix for high-resolution purification [78].

### 3.2. Scalability Considerations for Manufacturability Improvement

As part of manufacturability improvement, scalability assessment further ensures that the drug candidate, featuring the selected molecule format, is manufacturable by the newly developed cell line and process. Additionally, this new drug must be consistently producible across different scales with minimal parameter adjustments. This often includes tech transfer, facility fit analysis, and engineering runs, focusing on vessel geometry, mixing, and gassing. When lactate accumulation is higher and cell viability declines faster in large-scale cultures, optimizing agitation and oxygenation is usually the first step. Trace metal concentrations, particularly copper, may also need adjustment to prevent issues [79,80,81].

Despite significant efforts for process scaling-up, discussions on the scalability of cell lines for manufacturing are less common. Accelerated timelines for cell line development, driven by new technologies and automation, can lead to genetic instability and increased sensitivity to environmental stress [82], potentially inducing epigenetic modifications, lactate accumulation, and even cell death [81,83]. The addition of copper has been found to be effective in mitigating some of these issues but may not address all underlying causes.

Increased selection pressure during seed train expansion has been shown to improve production performance and scalability in CHO cultures without altering gene integration or copy number [84]. Lower lactate concentrations and enhanced re-maturation were observed, suggesting that re-cloning underperforming cell lines can enhance productivity and scalability.

## 4. Closing Remarks

B-cell-targeting biologics are innovative and successful new therapies for autoimmune diseases. While B-cell depletion therapy has shown efficacy in treating a range of autoimmune diseases, pan B-cell depletion can lead to broad immune suppression, increasing the risk of infections [85,86]. Further improvements in clinical efficacy and safety would therefore rely on continued efforts to discover new targets and to develop new molecule formats that specifically remove or inactivate pathogenic effector B cells while preserving regulatory B-cells for maintaining immune surveillance and minimizing adverse effects. While boosting potencies through avidity for deeper and more sustained effects, the bispecific format allows distinct targets to be simultaneously engaged and potentially increases the selectivity on certain populations of B-cells, such as autoantibodies-producing subpopulation, to avoid broad immune suppression. A bispecific format can also be applied in designing biologics that interact with two different cell types, such as B-cells and T-cells. T-cell mediated destruction of pathogenetic B-cells offers a different mode of action compared with the pan B-cell depletion through complement-dependent cytotoxicity and antibody-dependent cellular cytotoxicity; simultaneously targeting B-cells and engaging T-cells can help reset the immune system [17]. Product manufacturability needs to be evaluated as early as possible and iteratively with the inputs from process development teams. Selecting the right expression system and cell line based on the molecule format-specific needs provides an upfront advantage for maximizing titer while maintaining product quality attributes, which in turn simplifies later purification and formulation steps. Platform approach using standard mAb processes can be a good starting point, but it should be complemented with a well-characterized and molecule format-specific toolbox for further optimization to ensure high quality and cost-effective manufacturing. The toolbox contains various levelers of medium compositions including additives, resin selections, and in-process controls, but more importantly, it is empowered by deep mechanistic understanding. New technologies are on the horizon. We need continued learning, innovation, and exploration.

## Figures and Tables

**Table 1:** B-cell targeted biologics in autoimmune diseases and their molecule formats and features.

Target	Drug Name	Molecule Format/Features	Autoimmune Indications	References
CD20	Rituximab	Chimeric murine-human IgG1k mAb/targeting CD20 on pro-B cells and all mature B cells, but not long-lived plasma or plasmablast cells.	Approved: RA, GPA, MPA, PV Clinical trials: ITP, MG	[10]
	Ocrelizumab	Humanized mAb/with afucosylated glycoforms enhancing ADCC	Approved: RRMS and PPMS	[11]
	Ublituximab	Chimeric murine-human IgG mAb/with low-fucosylated glycoforms enhancing ADCC	Approved: RRMS, CIS, SPMS	[11]
	Ofatumumab	Fully human monoclonal antibody/first B-cell-targeting therapy that is intended for self-administration at home	Approved: RRMS, CIS, SPMS Clinical trial: RA	[11]
	Veltuzumab	Humanized mAb/epratuzumab framework and rituximab CDRs	FDA granted orphan status designation for ITP and pemphigus Clinical trial: RA	[7]
	TRU-015	Fully human IgG fusion protein/a single-chain Fv specific for CD20 linked to human IgG1 hinge, CH2, and CH3 domains but devoid of CH1 and CL domains	Clinical trials: active seropositive RA on a stable background of methotrexate	[12]
	Mosunetuzumab	Bispecific antibody/IgG, anti-CD20 and anti-CD3	Clinical trials: SLE	[13]
	Imvotamab	Bispecific antibody/IgM, anti-CD20 and anti-CD3	Clinical trials: RA, SLE	[13]
CD19	Inebilizumab	Humanized IgG1k mAb/with afucosylated glycoforms enhancing ADCC	Approved: NMOSD with AQP4-IgG+ Clinical trials: GM, IgG4-RD	[4,14] NCT04524273
	Obexelimab	Bispecific antibody/simultaneously binds CD19 and FcγRIIb, resulting in down-regulation of B cell activity	Clinical trials: GM; IgG4-RD	[15]
	Blinatumomab	Bispecific antibody/anti-CD19 and anti-CD3	Clinical trials: RA, system sclerosis	[16,17]
	PIT565	Trispecific antibody/anti-CD19, anti-CD3, and anti-CD2	Clinical trials: SLE	NCT06335979
CD22	SM03	Chimeric murine-human mAb/targeting the extracellular portion of CD22	Clinical trials: SLE, RA	[18]
	Epratuzumab	Humanized mAb/targeting CD22 with modest ADCC activity	Clinical trials: SLE	[19]
CD38	Daratumumab	Fully human mAb/targeting CD38 on long-lived plasma cells	Clinical trials: SLE	[15]
BAFF/BAFF-R	Belimumab	Fully human mAb/neutralizing biologically active soluble form of BAFF	Approved: SLE and lupus nephritis	[20]
	Ianalumab (VAY736)	Fully human mAb/antagonizing BAFF-R	Clinical trials: MS, SLE, Sjögren’s syndrome, Diffuse Cutaneous Systemic Sclerosis	[21]
CD40/CD40L	Dapirolizumab pegol	Fab/polyethylene glycol-conjugated, anti-CD40L, lacking the Fc-portion to avoid platelet activation	Clinical trials: SLE	[22]
	Iscalimab (CFZ533)	Fully human mAb/Fc-silenced, antagonizing CD40	Clinical trials: Graves disease (GD); Sjögren’s syndrome	[23,24]
	BI 655064	Humanized mAb/anti-CD40 blocking CD40-CD40L interaction	Clinical trials: RA	[25]
	Dazodalibep (AMG611, HZN-4920)	Ig-like scaffold-HSA fusion protein/Tn3 scaffolds derived from the 3rd fibronectin type III domain of human tenascin-C, structurally analogous to antibody CDRs and functionally blocking CD40-CD40L interaction	Clinical trials: RA, Sjögren’s syndrome	[26,27,28]
BAFF/APRIL	Telitacicept	Fc fusion protein/fused with extracellular domain (amino acids 13-118) of TACI binding to and neutralizing BAFF and APRIL	Approved: SLE (in China) Clinical trials: IgA nephropathy, MS, RA, MG, Sjögren’s syndrome	[29]
	Atacicept	Fc fusion protein/fused with extracellular domain (amino acids 30-110) of TACI binding to and neutralizing BAFF and APRIL	Clinical trials: SLE, RA, IgA nephropathy	[30]
CD32B/CD79B	PRV-3279 (MGD010)	Bispecific antibody/simultaneously targeting B-cell surface proteins CD32B and CD79B	Clinical trials: SLE	[31]

mAb = monoclonal antibody; RA = rheumatoid arthritis; GPA = Granulomatosis with Polyangitis; MPA = Microscopic Polyangitis; PV = Pemphigus Vulgaris; ITP = idiopathic thrombocytopenic purpura; MG = myasthenia gravis; GCA = giant cell arteritis; SSc-ILD = systemic sclerosis-interstitial lung disease; PJIA = polyarticular juvenile idiopathic arthritis (PJIA); SJIA = systemic juvenile idiopathic arthritis; MS = multiple sclerosis; CIS = clinically isolated syndrome; RRMS = relapsing–remitting MS; PPMS = primary progressive MS; SPMS = secondary progressive MS; IgG4-RD = IgG4-related diseases.

**Table 2:** CHO variant cell lines for afucosylated mAb productions.

Cell Line	Affected Biosynthesis Pathway	Reference
CHO Lec13 (Pro-Lec13.6a)	Natural deficiency in endogenous GDP-mannose 4,6-dehydratase (GMD)	[51,56]
CHO-DG44 FUT8^−/−^ (BioWa)	FUT8 knockout by homologous recombination	[57] Patent# US6946292B2
CHO-K1 FUT8^−/−^	FUT8 deletion by ZFN	[58]
CHO-gmt3 (CHO-glycosylation mutant3)	GDP-fucose transporter (SLC35C1) inactivation	[59]
CHO-RMD	Heterologous expression of GDP-6 deoxy-d-lyxo-4-hexulose reductase (RMD) in the cytosol of CHO cells to deflect the GDP-fucose de novo pathway	[60]
CHO-GnT-III	Overexpressed GnTIII catalyzes the formation of a bisecting GlcNAc to reduce Fc core fucosylation	[61]
CHO-SM	GDP-fucose 4,6-dehydratase (*GMD*) knockout, which makes the cell unable to produce intracellular GDP-fucose and fucosylated glycoproteins in the absence of L-fucose	[62]

## Data Availability

No new data were created.
